# Development of a Prognostic AI-Monitor for Metastatic Urothelial Cancer Patients Receiving Immunotherapy

**DOI:** 10.3389/fonc.2021.637804

**Published:** 2021-04-06

**Authors:** Stefano Trebeschi, Zuhir Bodalal, Nick van Dijk, Thierry N. Boellaard, Paul Apfaltrer, Teresa M. Tareco Bucho, Thi Dan Linh Nguyen-Kim, Michiel S. van der Heijden, Hugo J. W. L. Aerts, Regina G. H. Beets-Tan

**Affiliations:** ^1^Department of Radiology, The Netherlands Cancer Institute — Antoni van Leeuwenhoek Hospital, Amsterdam, Netherlands; ^2^GROW School for Oncology and Developmental Biology, Maastricht University, Maastricht, Netherlands; ^3^Artificial Intelligence in Medicine (AIM) Program, Mass General Brigham, Harvard Medical School, Boston, MA, United States; ^4^Department of Medical Oncology, The Netherlands Cancer Institute — Antoni van Leeuwenhoek Hospital, Amsterdam, Netherlands; ^5^Department of Biomedical Imaging and Image-guided Therapy, Medical University of Vienna, Vienna, Austria; ^6^Institute for Diagnostic and Interventional Radiology, University Hospital of Zurich, Zurich, Switzerland; ^7^Department of Molecular Carcinogenesis, The Netherlands Cancer Institute — Antoni van Leeuwenhoek Hospital, Amsterdam, Netherlands; ^8^Radiology and Nuclear Medicine, Maastricht University, Maastricht, Netherlands; ^9^Institute of Regional Health Research, University of Southern Denmark, Odense, Denmark

**Keywords:** artificial intelligence, immunotherapy, checkpoint inhibitors, urothelial cancer, treatment monitoring, imaging - computed tomography, response assessment, prognostication

## Abstract

**Background:** Immune checkpoint inhibitor efficacy in advanced cancer patients remains difficult to predict. Imaging is the only technique available that can non-invasively provide whole body information of a patient's response to treatment. We hypothesize that quantitative whole-body prognostic information can be extracted by leveraging artificial intelligence (AI) for treatment monitoring, superior and complementary to the current response evaluation methods.

**Methods:** To test this, a cohort of 74 stage-IV urothelial cancer patients (37 in the discovery set, 37 in the independent test, 1087 CTs), who received anti-PD1 or anti-PDL1 were retrospectively collected. We designed an AI system [named prognostic AI-monitor (PAM)] able to identify morphological changes in chest and abdominal CT scans acquired during follow-up, and link them to survival.

**Results:** Our findings showed significant performance of PAM in the independent test set to predict 1-year overall survival from the date of image acquisition, with an average area under the curve (AUC) of 0.73 (*p* < 0.001) for abdominal imaging, and 0.67 AUC (*p* < 0.001) for chest imaging. Subanalysis revealed higher accuracy of abdominal imaging around and in the first 6 months of treatment, reaching an AUC of 0.82 (*p* < 0.001). Similar accuracy was found by chest imaging, 5–11 months after start of treatment. Univariate comparison with current monitoring methods (laboratory results and radiological assessments) revealed higher or similar prognostic performance. In multivariate analysis, PAM remained significant against all other methods (*p* < 0.001), suggesting its complementary value in current clinical settings.

**Conclusions:** Our study demonstrates that a comprehensive AI-based method such as PAM, can provide prognostic information in advanced urothelial cancer patients receiving immunotherapy, leveraging morphological changes not only in tumor lesions, but also tumor spread, and side-effects. Further investigations should focus beyond anatomical imaging. Prospective studies are warranted to test and validate our findings.

## Introduction

Durable clinical benefit to immune checkpoint inhibitors in metastatic setting led to approval in several malignancies (1–3). Unlike traditional cancer treatments, such as chemotherapy and radiotherapy, which are administered for a predefined amount of time, immunotherapy is generally administered until there are tangible clinical benefits or until progressive disease/adverse events deem it unsuitable—for a maximum of 2 years. To achieve this, an accurate treatment evaluation method is required.

Whole-body Computed Tomography (CT) provides information on the full-picture of the patient. Beyond tumor size dynamics, CT imaging allows assessment of immune-related side-effects and/or disease-related complications.

Therapy response evaluation following CT is measured according to the *response evaluation criteria in solid tumor* (RECIST) (4), or iRECIST, adapted for immunotherapy (5). This involves prospective tracking of preselected lesions by measuring 2-dimensional diameters. Various immune-related toxicities and cancer-related complications that inform clinical practice may also be identified on CT scans, but are not accounted for in current RECIST criteria. So far, a comprehensive quantitative approach that involves quantitative response evaluation and clinically relevant conditions is lacking.

Quantitative approaches, such as radiomics, have been explored in the past (6, 7). While these led to satisfactory results in the field of prognostication, these rely mostly on manual segmentations, which are time-consuming and prone to human operator error. A comprehensive non-invasive method that comprises the assessment of tumor size dynamics and side-effects or other cancer-induced conditions, in an automatic and precise quantitative manner, would be preferable.

Novel techniques of computational imaging and artificial intelligence (AI) can be the basis for quantitative methods for treatment monitoring (8). Specifically, AI algorithms can be seen as methods to capture, measure, and quantify complex highly-variable anatomical phenomena for prognostic purposes, in a robust and time-efficient manner. To this end, we have developed an AI algorithm that performs automated tracking and quantification of morphological changes based on longitudinal CT imaging in immunotherapy treated patients, allowing correlations with overall survival. We term our AI system the *Prognostic AI-monitor* (PAM). Recently, a similar pilot approach was tested in a study on chest imaging of a NSCLC cohort (8), demonstrating accurate response prediction and a correlation with overall survival. In this study, we aim to extend the model to thoracoabdominal imaging, and validate it on a cohort of metastatic urothelial cancer patients treated with anti-PD1/PDL1. The model accuracy will be assessed at various time points within the treatment timeline, and the explicability through qualitative investigation of AI-generated prognostic heatmaps.

## Materials and Methods

### Study Cohort

We retrospectively included stage-IV urothelial cancer patients treated with anti-PDL1 or anti-PD1 monotherapy that had started follow-up imaging at the Netherlands Cancer Institute - Antoni van Leeuwenhoek hospital (NKI-AVL, Amsterdam, The Netherlands) between 07-2014 and 06-2018. Response evaluation was done using regular contrast-enhanced CT scans of the abdomen, chest, or both. For all patients, CT imaging scans acquired between 6 months prior to the start of immunotherapy, up to 2 years after, were collected. Inclusion criteria were high-resolution images (slice thickness ≤ 5 mm), and the presence of at least thorax or abdomen in the scan field. As we aim to use AI to track changes across follow-up, patients with <2 scans, at two different time points, could not be included. These criteria were verified automatically via the DICOM tag, or via the automatic localization algorithm proposed by Zhang et al. (9), respectively. For each patient, we recorded age at start of treatment, date of start of treatment, and date of death. Additionally, to compare PAM with current treatment monitoring standards, we collected parameters of radiological assessments (progression and response), as well as routine clinical blood analyses (hemoglobin, leukocyte count, thrombocyte count, and erythrocyte count). The entire dataset was divided into discovery and an independent test set based on the patients' ID (even IDs were assigned to the discovery set, odd IDs were assigned to the independent test set, creating a 50/50 split). The study was conducted at the NKI-AVL after approval of the local Institutional Review Board (IRBd19-083).

### Data Harmonization

A data harmonization protocol was applied to mitigate heterogeneity from typical real-world imaging datasets. This consisted of isotropic linear resampling of the scans at 2 mm, clipping of the Hounsfield units between−120 (fat) and 300 (cancellous bone), and rescaling of the intensities between 0 and 1. All images were cropped and padded to 192 × 192 × 192 voxels (160 axial coordinate for chest imaging).

### Prognostic AI-Monitor

PAM is composed of three AI-modules. The first module, termed *localizer*, consisted of a VGG-like convolutional network, tasked to crop out the chest and the abdomen in two separate images, each according to standardized anatomical locations. These were defined as the space between the lower neck and the lower diaphragm, and the space between the upper diaphragm and the lower pelvis, respectively. The second and third modules, termed *tracker*s, consisted of two instances of the same convolutional network, one trained for chest imaging, and one for abdominal imaging, tasked to quantify morphological changes between pairs of images. We termed these modules the *chest* and *abdominal tracker*, respectively. Their architecture was based on radiological deep learning-based image-to-image registration. At its core, each tracker is tasked to match anatomical landmarks and shapes of two 3D radiological images. In doing so, the network learns to quantify anatomical differences between pairs of scans. We leveraged the tracker network knowledge (i.e., its *latent representation*) to extract quantitative imaging feature vectors representing morphological changes between follow-up scans of the same patient, and fed them into a classifier trained to predict survival. Time from start of treatment, and time between scans were also fed into the classifier, for temporal reference.

### Localizer Module

The localizer module was designed following the research of Zhang et al. (9). The authors showed how a convnet trained to sort slices in a specific order (e.g., from head to toe) can be used for anatomical localization. The network followed a siamese learning scheme. It received a pair of CT slices from a single scan, and had to learn which of the two slices would be on top of the other in the original CT scan. The only way for the network to learn to perform this task would be for the network to assign to each anatomical location a number *i* that would increase from head to toe. Once the training was complete, we used the network to retrieve a specific location by searching for their assigned number (for example, in our case, the upper-most point of the diaphragm was always assigned to be around *i* = *25*). This algorithm idea is particularly powerful, as the ground truth (i.e., the order of the slices) can be automatically extracted from the CT scan, and therefore it does not require any manual labeling.

**Figure 1:**
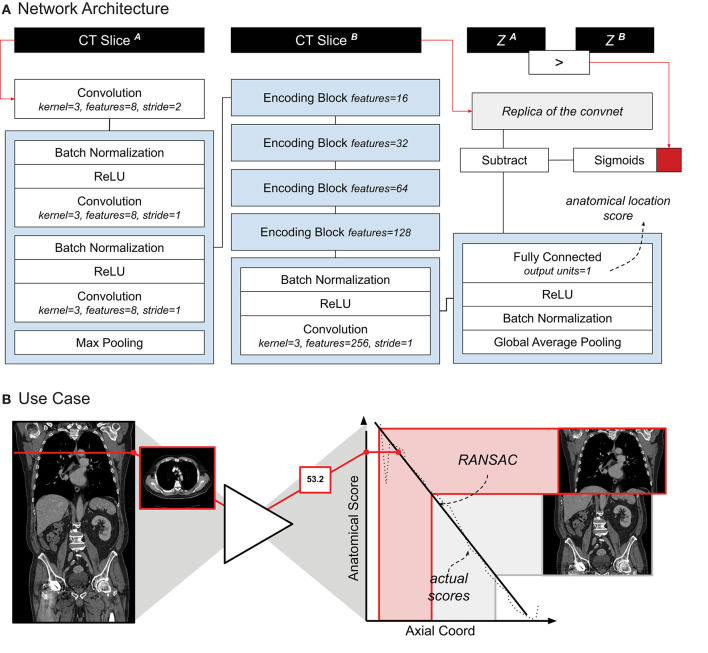
**(A)** Schematic representation of the localizer architecture and its training. The CT slices in input are axial slices taken from the same CT scan. Z^1^ and Z^2^ are the axial coordinates of each input slice, respectively. The red square symbolizes the binary cross entropy loss function used during training. **(B)** A use-case of the localizer. Each axial slice is processed through the network to generate a score. A linear relation between the scores and the axial coordinate is estimated. Cropping of the thorax and abdomen is done based on the anatomical scores, and corresponding axial slice.

Our localizer module was built largely based on Zhang's architecture design — the exact architecture we used is shown in Figure 1A. The network was trained following the same siamese learning scheme of the original research (9). Binary cross-entropy was the loss function chosen, the optimizer was Adam with an initial learning rate of 0.001, and the batch size was set to 8 (i.e., 8 random scans, one random pair of slices per scan). As it was difficult to set a number of epochs (considering it could be based either on the number of scans or on the number of slices), we chose to set a general number of iterations, namely 50,000. RANSAC regression (10) was used to model the relation between the network score and the actual slice number for each scan. We chose RANSAC for its robustness to irregularities provided by the localizer algorithm. Figure 1B shows the localizer network applied to a scan.

### Tracker Module

The tracker module was designed following the research of Balakrishnan et al. (11) and Zhao et al. (12), as well as our previous work on chest imaging in NSCLC (8). The network receives two images as input (i.e., a moving and fixed one) concatenated along the channel axis. The architecture of the network processes the input in two subsequent parts. The first part, consisting of VGG-like convnet, parses the images through a series of five subsequent convolutional blocks and two fully connected layers, to regress the 12 parameters of the affine transform. This is used to give a linear pre-alignment between the input images, correcting for different patient positions. The second part of the network follows a U-Net architecture (13), where the inputs (i.e., the affine warped moving image and the fixed image) are processed together to regress a displacement field. The displacement field specifies for each voxel a 3D vector. The vector indicates where the voxel in that location of the moving image would be displaced to, in order to match the corresponding anatomical structure in the fixed image. This part of the network consisted of an encoder with four convolutional blocks downsampling the images by half the size via striding, a convolutional latent space with stride of one, and four deconvolutional blocks each upsampling the inputs by double the size via striding. Skip connections were implemented between encoding and decoding layers following the implementation in the original paper. Both affine and deformable parameters are applied to the moving image through a spatial transformation layer.

**Figure 2:**
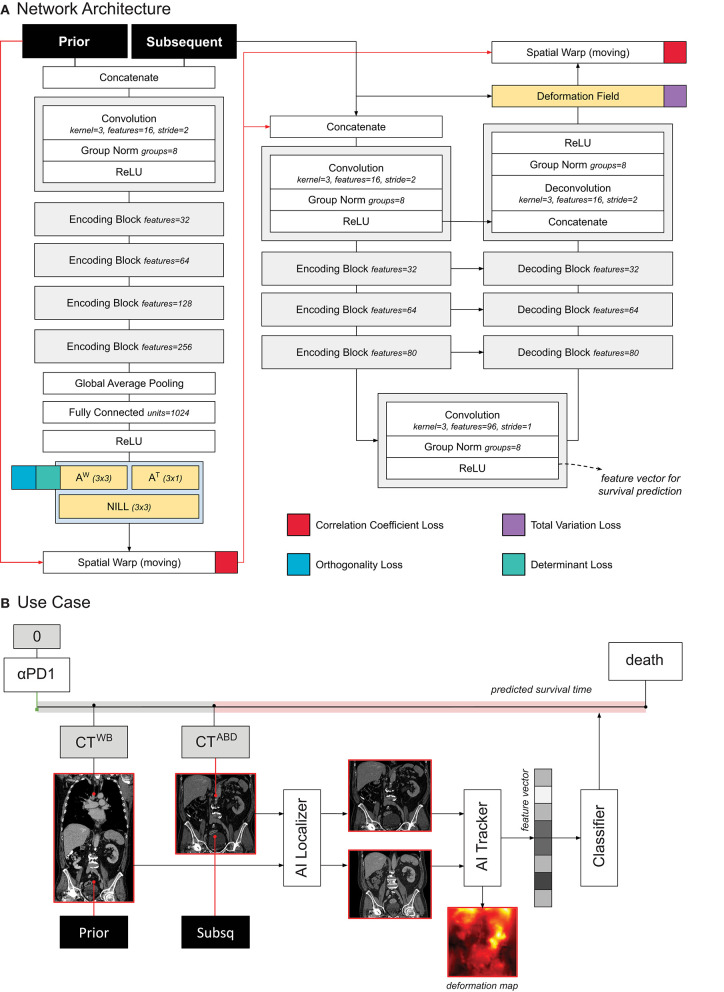
**(A)** Schematic representation of the tracker architecture and its training. **(B)** Use case of the tracker within PAM.

The network was trained to minimize the correlation coefficient loss (11, 12). Three penalties were also employed to mitigate for unlikely morphological deformations: two on the affine loss (weighted 1/10), and one on the deformable loss (weighted 1/100). We decided to decrease the weight on the deformable loss to give to the model more freedom in modeling abdominal changes. Adam optimizer was used during training, with an initial learning rate of 3 × 10^−4^. A curriculum learning scheme was implemented during training, such that the loss would be computed on a decreasingly smoother version of the images. The smoothing was implemented via average pooling, starting with a kernel size of 9, and reduced by 3 at epochs 100, 150, and 175. Batch size was set to 2. To mitigate negative effects resulting from the small batch size, group normalization was employed instead of batch normalization (14). Figure 2A shows a detailed overview of the model and the loss used.

Both the localizer and trackers were unbiased toward both cancer and treatment, and could be trained on unlabeled data. Using The Cancer Imaging Archive (TCIA) (15), we collected^1^ all available radiological images, and excluded scans with non-axial acquisition, low resolution (> 5 mm), animals (e.g., mice, suine) and phantoms. Based on thorax-abdominal CT scans, we then trained the localizer module on a lymphadenopathy dataset and extracted abdomen slices from all archived CT scans. Next, the isolated set of abdominal CT scans (16–35) were employed to train the abdominal tracker PAM module. We kept a 10% hold out during training to control for overfitting (i.e., patients whose ID were multiples of 10 were held out). At each training iteration, we created a batch by randomly sampling pairs of TCIA's abdominal CT scans. This implies that the network learned, in principle, to register pairs (likely) composed of scans from different patients, or even different datasets. This auxiliary task represents a more complex problem than the one of matching follow-up scans of the same patient. Our goal was for the AI to learn to match corresponding imaging landmarks, and to cover a large set of possible variations while, simultaneously, containing its scope (or *prior knowledge*) to landmarks in the abdominal area. For the chest AI tracker module, we leveraged the trained weights from the NSCLC study (8). The code of both tracker and localizer have been added to the department AI repository^2^.

### Association With Survival

In order to predict survival, we trained a logistic regression classifier based on the quantitative features extracted from the tracker. More specifically, we leveraged the feature maps in the deepest layer of the U-Net (this is shown in Figure 2A). To obtain a feature vector that can be used for the standard logistic classifier, we applied global average pooling. The resulting feature vector (96 entries or features) was fed into the logistic regression model to predict whether the patient would die within 1 year after the date of the latter scan, see Figure 2B. For simplicity, the higher resolution information flow in the skip layers and in the final deformation field were not utilized for prognostication. Time from start of treatment, and time between scans were also fed into the classifier, for temporal reference. For each patient, we employed any two scans that were at most 1 year apart from each other.

### Comparison to Clinical Standards for Monitoring

We compared PAM against radiological assessments and blood values. For simplicity, we limited the analysis to PAM-scores of abdominal imaging. We employed both univariate and multivariate comparison. The large majority of scans included in the analysis of PAM did not have a corresponding radiological assessment, or blood exam done on the same day — in other words, there was no one-to-one matching for the majority of the cases. To overcome this limitation, we averaged the values of both radiological assessments and blood work over a window of 6 weeks, centered on the date of the CT scan analyzed by PAM. Since PAM leverages tracking of morphological changes, we applied the same principle to the blood values. Namely, we estimated the rate of change of each blood value over time, i.e., *(v*_*s*_ –* v*_*p*_*)/dt*, where *v*_*p*_ and *v*_*s*_ is the blood values at prior and subsequent scan, respectively, and *dt* is the time in between.

Radiological progression and response were assessed based on an increase in diameter of 20% or decrease of 30% in diameter, respectively, according to RECIST standards. Diameters were derived using *d* = $\sqrt[{3}]{}$*(6V/*π*)*, where *V* is the tumor volume delineated by a radiologist (PA). As these assessments already represented longitudinal change, they were left untouched, allowing for the creation of two classes (i.e. “response” and “progression”).

### Prognostic Heatmaps

In order to interpret the results from PAM, we employed an occlusion sensitivity method (36). With this method, we occluded a section (or patch) of the image to the AI, by setting its voxel intensities to zero. We collected the prediction made by the AI on the occluded image, and compared it with the prediction on the original image. The importance of that patch was defined as the absolute difference between the predictions made on the occluded and the prediction made on the original image. A heatmap was generated by scrolling the occluded patch through the image, and collecting the relative importance of each patch. We termed the resulting visualization the prognostic heatmap. The pseudo-code of the algorithm is presented in Algorithm 1. A board-certified radiologist (TNB, specialized in thoracic and abdominal oncologic imaging, blinded to the outcome) was tasked to visually analyse the prognostic maps for a subcohort of the validation set. These were patients that had both thoracic and abdominal imaging. We chose the first available scan pair closest to the start of treatment — namely baseline and first follow-up. The radiologist was tasked to identify the location of highlights on the heatmaps, as well as pathologies/anomalies that were not highlighted, i.e., “hotspots” and “coldspots,” respectively. Expert assessments were categorized based on whether they were hotspots or coldspots. This resulted in three classes of interest: hotspots on tumor lesions or therapy-related lesions, hotspots on seemingly healthy parenchyma, and coldspots on tumor or therapy-related lesions. Coldspots on healthy tissues are trivial, and therefore not accounted for.

**Algorithm 1:** **A1**. Generation of Heatmaps for Model Explainability.

Input (prior, subsequent, time_start, time_delta)	
1	Reference_score ← PAM(prior, subsequent, time_start, time_delta)
2	ROI ← (0:64, 0:64, 0:64) *[A]*
3	Occluded_prior, Occluded_subsequent ← copy (prior), copy (subsequent)
4	Occluded_prior[ROI], Occluded_subsequent[ROI] ← 0, 0
5	Occluded_score ← PAM(occluded_prior, occluded_subsequent, time_start, time_delta)
6	ROI_importance ← |occluded_score - reference_score|
7	Prognostic_map[ROI] ← maximum (prognostic_map[ROI], roi_importance) *[B]*
8	Move the ROI 8 voxels along one of the axis
9	If ROI has not scrolled through the whole image yet, go to Step 3
10	Return prognostic_map

*[A] cube of 64 × 64 × 64 in the top left back corner, [B] since the ROI are overlapping, we chose to use the maximum function, which prevents erroneous overriding of previous estimation*.

### Statistical Analysis

PAM aims to predict whether the patient will die within 1 year after the date of the latter scan. As this is done through a classification system, we evaluated the performance of the model using classical classification statistics. Namely, we assessed specificity, sensitivity, and area under the receiver operating curve (ROC-AUC). Statistical significance was assessed using the Mann-Whitney-U test. Confidence intervals were estimated via bootstrapping performed using sampling with replacement (1,000 times). Statistical comparison between ROC-AUC was performed via McNeils' test. Multiple hypothesis testing was corrected with the false discovery rate (FDR) method with alpha set at 10%. A generalized multivariate linear regression was employed to evaluate the significance of PAM against current clinical standards (radiology and blood work).

## Results

### Study Cohort

**Figure 3:**
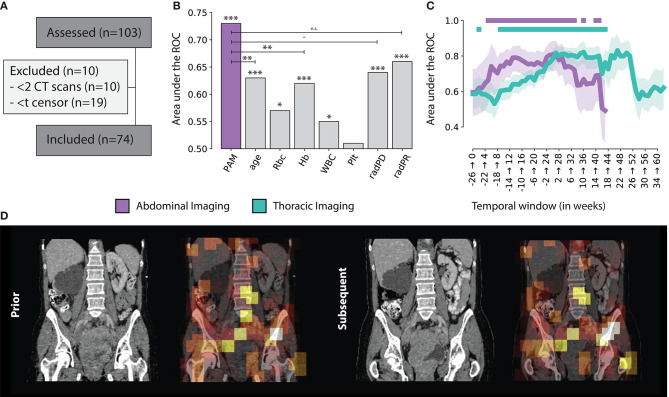
**(A)** Consensus, **(B)** PAM Abdominal tracker performance compared to other standard factors used for treatment monitoring. Significance levels are reported for *p* < 0.001 (***), 0.01 (**), 0.05 (*), 0.1 (·) and n.s. for non-significant **(C)** PAM Abdominal and Thoracic monitoring performance, over time, with respect to the start of treatment, in weeks. The | indicates statistical significance after FDR correction **(D)** Example of the prognostic heat map overlaid on top of the original abdominal scan.

A total of *N* = 103 patients were included in this study. Ten patients had only one scan available, making it impossible to model longitudinal changes, and therefore had to be excluded from the analysis. Nineteen patients did not have enough time between imaging date and censor date, and were excluded (see Figure 3A). The median age in this cohort was 65 years (IQR: 55 — 72). Upon stratification, *N* = 37 patients were assigned to the training set, and *N* = 37 in the validation set. In terms of overall survival, the median was reached in about 1 year (345 days).

Imaging-wise, we included a total of *N* = 1,087 CT scans between 6 months before start of treatment and up to 2 years after. These were used to create the scan pairs needed for PAM to model morphological changes. In total, we found *N* = 2,339 abdominal, and *N* = 7,431 chest scan pairs. We further excluded all scan pairs of living patients whose time between the latest scan and censor was <1 year, and whose time between scans in the scan pair was more than 1 year. This resulted in *N* = 1,209 abdominal scan pairs, and *N* = 3,701 chest scan pairs in the discovery set and *N* = 614 and *N* = 1,937, in the validation set, respectively. We chose not to limit the analysis to only subsequent scans, as the time points of when they were taken, and the time interval between them might vary. We rather chose to include all feasible pairs, within a given time-interval.

With respect to the unlabelled data used for training, we retrieved a total of *N* = 37,573 CT scans from TCIA. The localizer was trained first, on *N* = 176 thoracoabdominal CT scans from the lymphadenopathy dataset. The abdominal tracker was trained on *N* = 3,137 abdominal CT scans, resulting from the automatic inclusion procedure.

### Prognostic Performance

We assessed the ability of the classifier (trained on the imaging features of the tracker module) to predict 1 year survival after the latter scan of the scan pair. Across all scan pairs and treatment course (up to 6 months before and 2 years after start of therapy), the overall performance on the independent validation set was 0.73 AUC (CI: 0.69–0.76, *p* < 0.001) for abdominal images, and 0.67 AUC (CI: 0.64–0.69, *p* < 0.001) for chest images. Specificity and sensitivity were 0.74 (CI: 0.69–0.80) and 0.60 (CI: 0.56–0.64) for abdominal images; and 0.71 (CI: 0.68–0.74) and 0.58 (0.56–0.60) for chest images, respectively.

This results gives us an overview of the performance of PAM, independently from the treatment time point or interval between follow-up scans. To gain additional insights in the performance of PAM at different treatment time points, we employed a temporal analysis of the performance on a 6-months moving window. Particularly, we estimated the performance of PAM for all pairs acquired between day *d* and *d* + 6 months, with *d* moving forward by 7 days at each step. The analysis was run on temporal windows with at least 10 positive and 10 negative samples to limit statistical noise. For abdominal scans, the highest prognostic performance was reached in the first 6 months of treatment (7–189 days), with an ROC-AUC of 0.82 (CI: 0.72–0.89, *P* < 0.0001). In general, the temporal windows around and up to the first 8 months of treatment seem to be the ones carrying the highest predictive value, staying significant after correction for multiple hypothesis testing. Similar results were obtained for chest scans. The highest prognostic performance was reached later than the abdominal model, around 5–11 months after start of treatment, with a ROC-AUC of 0.83 (CI: 0.71–0.92, *P* = 0.0002). Unlike abdominal scans, which were observed to have a prognostic value both around and during treatment, chest scans carried much higher prognostic value during treatment rather than around the start date. Detailed results of the prognostic performance over time are shown in Figures 3B,C.

To investigate PAM as a biomarker, we analyzed the scans taken before the start of treatment. Namely, we investigated the abdominal scan pairs whose scans were taken between 12 weeks prior and start of treatment. This resulted in 31 scan pairs of 26 patients in the external validation set. Four patients had multiple scan pairs. We aggregated multiple scan pairs per patient by taking the average PAM prediction. This resulted in an AUC of 0.70 (CI: 0.50–0.88, *p* = 0.054) for the prediction of 1 year survival from the moment of start of treatment. Specificity and sensitivity were 0.69 (CI: 0.50–0.87) and 0.82 (CI: 0.58–1.00), respectively. Further analysis on PAM predicted survival at baseline showed a significant difference of >464 days between low- and high-risk patients (*p* = 0.012, *log rank test*), with the high-risk group had a median survival of 266 days, and the low-risk group did not reach median survival within the first 2 years of treatment. Combination of the abdominal and thoracic scores (i.e., average) yielded a lower performance in terms of classification (0.65 AUC, CI: 0.46–0.83, *p* = 0.118), and virtually unchanged results for the survival analysis (*p* = 0.012, *log-rank test*). The limited number of patients did not allow to explore more advanced methods for score combinations.

### Comparison With Current Monitoring Standards

Univariate analysis for current monitoring standards showed significant performance for both radiological assessments, as well as laboratory (hemoglobine, erythrocytes, leukocytes, and thrombocytes) results. Radiological progression and response reached an AUC of 0.64 (CI: 0.58–0.70, *p* < 0.001) and 0.66 (CI: 0.62–0.69, *p* < 0.001), respectively. In terms of blood markers, increases in erythrocyte counts (0.57 AUC, CI: 0.51–0.62, *p* = 0.019), hemoglobin (0.62 AUC, CI: 0.57–0.66, *p* < 0.001), and leukocyte counts (0.55 AUC, CI: 0.49–0.61, *p* = 0.039) were all significant. None of these markers performed better than PAM. PAM performance remained statistically significant against these other biomarkers using multivariate analysis. Other factors that retained significance were radiological progression (*p* < 0.001), leukocyte count (*p* = 0.023), and age (*p* = 0.006). Hemoglobin and erytrocyten were displayed with a high correlation (0.92) and were averaged together. Their average, along with radiological response and thrombocyte count were not significant. Results of both univariate and multivariate are presented in Table 1 and Figure 3B.

**Table 1:** Prognostic performance of PAM against current monitoring tools.

**Univariate analysis**					
	**N. negative/positive**	***p*****-value**	**ROC AUC (95 CI)**	**Sensitivity**	**Specificity**
Erythrocyte count (Δ/dt)	358/110	**0.019**	0.57 (0.51–0.62)*	0.47 (0.43–0.52)	0.42 (0.34–0.49)
Hemoglobin (Δ/dt)	372/122	**<** **0.001**	0.62 (0.57–0.66)*	0.47 (0.43–0.51)	0.38 (0.31–0.45)
Leukocyte count (Δ/dt)	366/116	**0.039**	0.55 (0.49–0.61)	0.52 (0.48–0.56)	0.56 (0.49–0.64)
Thrombocyte count (Δ/dt)	366/116	0.421	0.51 (0.45–0.56)	0.51 (0.47–0.56)	0.54 (0.46–0.61)
Radiological Progression	145/65	**<** **0.001**	0.64 (0.58–0.70)	0.87 (0.82–0.91)	0.42 (0.31–0.52)
Radiological response	145/65	**<** **0.001**	0.66 (0.62–0.69) ^*^	0.69 (0.62–0.75)	0.00 (0.00–0.00)
AI-score (abdomen)	437/117	**<** **0.001**	0.73 (0.69–0.76)	0.60 (0.56–0.64)	0.74 (0.69–0.80)
AI-score (thorax)	1,421/516	**<** **0.001**	0.67 (0.64–0.69)	0.58 (0.56–0.60)	0.71 (0.68–0.74)
**Multivariate analysis**					
	**Coefficient**	**Standard deviation**	**95 Confidence interval**		***p*****-value**
Intercept	−1.1010	3.213	−7.398	5.196	0.732
AI-score (abdomen)	−7.9394	1.683	−11.239	−4.640	** <0.001**
Age	−7.3906	2.699	−12.680	−2.101	**0.006**
Erythrocyte + hemoglobin (Δ/dt)	−0.2210	2.455	−5.034	4.592	0.928
Leukocyte count (Δ/dt)	10.9735	4.810	1.546	20.401	**0.023**
Thrombocyte count (Δ/dt)	−0.3935	2.022	−4.357	3.570	0.846
Radiological progression	−3.0030	0.693	−4.361	−1.645	** <0.001**
Radiological response	> 100	> 100	< −100	> 100	0.999

AUC <0.5 were inverted for readability, indicated by *.

### Visual Analysis of Abdominal Heatmaps

Results from visual analysis were classified based on highlighted areas (hotspots), and whether they were cancer lesions, cancer-spread complications, therapy-induced complications, or seemingly healthy tissue. If cancer lesions and cancer-spread or therapy-induced complications were not covered by a hotspot, these were flagged as coldspots. In total, *N* = 31 cases were analyzed. Table 2 shows a summary of the results. A heatmap example is shown in Figure 3D.

**Table 2:** Visual analysis of PAM generated prognostic maps.

	**Rare (<10%)**	**10–25%**	**25–50%**	**Frequent (>50%)**
**Abdominal imaging**				
Hotspot tumor	Lung mets (3), adrenal mets (1), abdominal wall mets (1), ureter (1), recurrence (2), deposition (2)	Bone mets (6), peritoneal (5)	Bladder Ca (11), lymph nodes mets (16), liver mets (8)	
Hotspot tumor-related	Ascites (2)	Hydronephrosis (4)		
Hotspot therapy-related				
Hotspot healthy	Pelvis (2), genital (3), retroperitoneum (2)	Chest wall (7), pancreas (6)	Abdominal wall (14), stomach (13)	Bowel (21), liver (21), spleen (17), kidneys (18), spine (26), pelvic bone (27), hip region (28)
Coldspot tumor	Bladder Ca (2), lymph nodes mets (3), lung mets (1), bone mets (1), liver mets (2), peritoneal mets (2), abdominal wall mets (1), recurrence (1)			
Coldspot tumor-related	Pleural effusion (1), ascites (1), hydronephrosis (1)			
**Chest imaging**				
Hotspot tumor	Lymph nodes mets (3), bone mets (2)	Lung mets (7), liver mets (4)		
Hotspot tumor-related	Pleural effusion (1)			
Hotspot therapy-related	Pneumonitis (1), sarcoid like (2)			
Hotspot healthy	Lung (2)			Mediastinum (25), chest wall (27), upper abdomen (22), spine (26)
Coldspot tumor	Lymph nodes mets (3), lung mets (4), bone mets (2), liver mets (1)			
Coldspot tumor-related	Pleural effusion (1), ascites (1)			

*Ca, cancer; Mets, metastases. Number of cases between parenthesis (N)*.

In the abdomen, primary bladder tumors (*N* = 13), involved lymph nodes (*N* = 18) and liver metastases (*N* = 10) were flagged as prognostic by the AI algorithm in most cases where they were present—namely, hotspots in 85, 83, and 80% of cases, respectively. Similar frequencies were observed for bone (*N* = 7) and peritoneal metastases (*N* = 7), having been flagged in 86 and 71% of cases. Rare occurrences of adrenal metastasis, as well as abdominal wall metastasis and a ureter mass were also found, both as hotspots and coldspots. Low occurrence was also observed for cancer spread-related complications. These were hydronephrosis (*N* = 5), ascites (*N* = 3) and pleural effusion (*N* = 1). Hydronephrosis and ascites were highlighted in 4 and 2 cases, respectively. Far more common were hotspots observed on seemingly healthy tissue, including the hip region (*N* = 27), pelvic bone (*N* = 26), spine (*N* = 25), liver and bowels (*N* = 20), kidneys (*N* = 17), and spleen (*N* = 16). It was further observed that, in the large majority of cases, only part of the tissue would be highlighted, but never the full organ.

### Visual Analysis of Chest Heatmaps

In the thorax, 7 out of 11 lung lesions were highlighted (64%). The mediastinum, chest wall, and upper spine were the most common hotspots in seemingly healthy areas. Other lesion types, such as lymph node metastases and bone metastases, were also present but low in numbers. Observed cancer spread-related complications include pleural effusion (hotspot in 1 out of 2 cases), and ascites (coldspot). Pneumonitis and sarcoid-like disease were also present as therapy-related complication hotspots, but both as single cases. A summary of the results is shown in Table 2.

## Discussion

Advanced and non-invasive imaging methods for evaluation of treatment response, which would provide comprehensive and reliable information on how the patient responds to treatment, could improve accurate clinical decision making. Our aim was to assess the prognostic value of AI-enriched thoraco-abdominal CT response assessment in stage-IV urothelial cancer patients undergoing immune checkpoint inhibitors. We set up a fully-automatic AI-system that would track changes between follow-up thoraco-abdominal CT scans, and linked their quantitative descriptors to overall survival. We term this method *prognostic AI-monitor* (PAM).

Our findings showed that PAM reached significant predictive performance for both thoracic and abdominal CT, with AUCs of 0.67 and 0.73, respectively, for the prediction of 1-year overall survival from the moment of the scan. In-depth analysis revealed stark differences in the prognostic value of morphological changes depending on the time point of treatment, with the first 9 months of treatment being the most predictive and significant AUCs > 0.70, peaking to over 0.80 for both abdominal imaging, and thoracic imaging. Similar findings were observed in our previous study on NSCLC (8), where the changes recorded by the algorithm in the first 3 to 5 months of treatment were observed to have a higher prognostic value. In the present study, we extended the system to include both the thorax and abdomen, and trained with far larger datasets both in terms of pre-training as well as survival association. The AI algorithm designed in this study was significantly extended to a comprehensive AI-system (i.e., PAM), able to scan imaging data, identify the regions of interests, and analyze them for the purpose of monitoring and prognostication. By including abdominal images, we also showed that the previous system (8) can be extended to multiple parts of the human body.

To the best of our knowledge, this is one of the first studies employing artificial intelligence for prognostication in immunotherapy-treated urothelial cancer patients. In the study by Park et al. (37), the authors developed a radiomics model for the prediction of objective response and overall survival in a similar population. Machine learning was also employed on imaging (radiomics) features, however these were extracted via manually delineated lesions. The authors reported an AUC of 0.88 (CI: 0.65–0.97) for objective response prediction of bladder tumors in a cohort of *N* = 21 patients, with a significant difference in overall survival between (radiomics-identified) higher and lower risk groups. Our findings also showed significant differences in survival, but in contrast to the above study, we looked at the whole body changes, not only those of the tumoral lesions but also the non-tumoral treatment- or cancer-related changes (e.g., side-effects, organ compression, etc.). Our results are comparable to state-of-the-art methods based on time-consuming, error-prone, manual delineations (6, 7). Till now, single lesion analysis has allowed the field to develop, however, it has restricted the usage of the image only to selected areas-of-interest, accounting for <5% of the total data in the scan. While these methods have been refined to leverage known factors in cancer growth, including vascularity (38), oxygenation (39), and metabolic activity (40)—our approach is different. Not only do we offer a novel fully automatic procedure which completely eradicates the need of time-consuming segmentations, but it also makes use of the whole body image of the patient, to evaluate the patient's status and estimate survival.

We analyzed the PAM further, by means of visualization. More specifically, we employed a visualization method (36) to generate heatmaps, which highlighted regions of the image that carried higher predictive value, according to PAM. In our case, hotspots would correspond to gross morphological changes that the AI algorithm deemed of prognostic relevance. An expert radiologist was tasked to visually confirm these findings. Our findings show that changes in the primary tumor of the bladder, as well as metastases in lymph nodes, liver, peritoneum, and skeleton were among the most predictive for the algorithm.

Interestingly, there are similarities between our results, and the results from the NSCLC study (8). In both cases, the region of the primary tumor, as well as lymph nodes and bone lesions were closely inspected by the algorithm. Additionally, in the present study, the algorithm is also tracking changes in liver and peritoneal metastases. Unlike the present study however, the AI in the NSCLC cohort was working only on chest imaging, therefore unable to access the abdominal cavity.

There is evidence, in both studies, that bone lesions should be accounted for in imaging evaluation schedules. These are considered non-target lesions in the current response criteria and are notoriously difficult to assess (4, 5, 41). Both bladder and lung cancer generated evidence to support the further investigation for the inclusion of CT changes in the bone among the target lesions.

Generally speaking, these findings suggest an unequal effect of cancer lesions on survival. While this might seem trivial at first (e.g., brain metastases are known to have worse prognosis), all current imaging methods for response evaluation and prognostication [like the RECISTs (4, 5, 41)] do not distinguish between lesion types. RECIST methods are based on the change in the sum of diameters of a (limited) set of lesions. In other words, the growth of lesions in one organ is measured and weighed in the same way as the growth of another lesion in a different organ—no distinction is made. Our results however suggest that these factors should be accounted for, which would therefore require a more comprehensive evaluation scheme.

In this study, we proposed a method that is based on image-to-image registration, leveraging the properties of this technique in finding corresponding anatomical landmarks in pairs of images, and therefore constructing a model able to track not only tumors but also tumor- and therapy induced changes, as well as seemingly healthy parenchyma. Our method does not preclude the usage of other techniques and methods. As we have observed, commonly used clinical response evaluation tools also retained significance when compared against PAM, suggesting PAM as a complementary value to the current clinical standards. An optimal approach to the utilization of PAM would be integration of this method with other diagnostic tools currently available (42).

The study was limited to whole-body CT, which is the workhorse in standard clinical practice. As brain imaging is not part of the standard whole-body CT protocol, anatomical and functional changes in the brain as captured on MRI and PET/CT are yet to be explored. We envision a more comprehensive usage of this technique, where all available imaging during follow-up is leveraged for prognostication purposes. It is also yet to be confirmed whether the PAM approach would extend to other treatments and cancer types, and to which extent survival associations would be interchangeable. Further development of PAM should also focus on pre-treatment scans. In this study, we analyzed scans acquired up to 6 months before the start of treatment. Some patients had to be excluded, as they lacked follow-ups. We acknowledge that this could have introduced a bias toward patients with extensive treatment history, or against patients with the worse survival outcomes. An extension of PAM to include more of the treatment history would be beneficial in this sense, but it would require PAM to deal with the plethora of all different treatments, and combinations thereof, that nowadays oncological patients receive, hence beyond the scope of this study.

Another limitation of the study is the monocentric nature of the analysis. While CT data is generally acknowledged to have higher level of reproducibility across vendors than MRI, it is yet to be seen whether this would hamper the association with survival, and to what extent. Nonetheless, we made sure to train the tracker and localizer modules on large publicly-available datasets that would, in theory, provide a larger pool of variations in image acquisition protocols. Future studies should be focused on including a larger cohort of patients. This would allow not only to increase the number of features used in deformation modeling (now limited to 96), but also the machine learning classifier used for predicting survival, which could in turn increase the performance of the model.

Finally, the readers were, just like the algorithm, blinded to the patient's full-history. This did not allow them, for example, to perform a complete RECIST assessment, which would require the computation of a nadir. Further investigations should also focus on a full-comparison of PAM and RECIST criteria (and iRECIST), on whether they are complementary or mutually exclusive, and what are the benefits of using one or the other.

As a future outlook, we envision an extended PAM-like algorithm to be set up as a clinical decision support system in tumor boards, providing continuous monitoring and prognostication information, in order to assist physicians in the treatment decision process.

## Conclusions

In this study, we investigated the prognostic information of AI-derived whole-body imaging monitoring markers in advanced urothelial cancer receiving checkpoint inhibitors. We hypothesized that quantitative AI-derived features describing morphological changes happening during the course of treatment could hold prognostic information. To this end, we designed and implemented a prognostic AI-monitor (PAM). Our findings demonstrate that PAM is complementary to existing monitoring methods, while reaching comparable or superior accuracy. We argue that this could be the result of PAM's ability to analyze the whole body, including non-target cancer lesions and non-cancer lesions. Further investigation should focus on the development of a comprehensive pipeline beyond anatomical imaging, as well as on external validations.

## Data Availability Statement

The datasets presented in this article are not readily available because local privacy regulation do not allow us to share CT scans publicly for this project. Feature sets are available on request. Requests to access the datasets should be directed to ST, s.trebeschi@nki.nl.

## Ethics Statement

The studies involving human participants were reviewed and approved by Institutional Review Board, NKI-AVL. Written informed consent for participation was not required for this study in accordance with the national legislation and the institutional requirements.

## Author Contributions

ST: software development. ST, ZB, TB, TT, and TN-K: conceptualization and experimental design. ZB, TB, and ND: clinical results validation and interpretation. ST, ZB, TN-K, and ND: radiological and clinical data collection and curation. MH, HA, and RB-T: project supervision and resource acquisition. All authors: results, manuscript editing, and validation.

## Conflict of Interest

MH has consultancy agreements (paid to the institute) with Roche Genentech, BMS, Merck, and AstraZeneca; and grants (paid to the institute) from BMS, AstraZeneca, and Roche. The remaining authors declare that the research was conducted in the absence of any commercial or financial relationships that could be construed as a potential conflict of interest.

## Footnotes

^1^Accessed on the 21st of April 2020.

^2^Github: github.com/nki-radiology/PAM.
